# Variation of Serine-Aspartate Repeats in Membrane Proteins Possibly Contributes to *Staphylococcal* Microevolution

**DOI:** 10.1371/journal.pone.0034756

**Published:** 2012-04-11

**Authors:** Jing Cheng, Huping Xue, Xin Zhao

**Affiliations:** 1 Department of Animal Science, McGill University, Montreal, Quebec, Canada; 2 State Key Laboratory of Genetic Engineering, School of Life Sciences, Fudan University, Shanghai, China; University of Massachusetts Medical School, United States of America

## Abstract

Tandem repeats (either as microsatellites or minisatellites) in eukaryotic and prokaryotic organisms are mutation-prone DNA. While minisatellites in prokaryotic genomes are underrepresented, the cell surface adhesins of bacteria often contain the minisatellite SD repeats, encoding the amino acid pair of serine-asparatate, especially in *Staphylococcal* strains. However, their relationship to biological functions is still elusive. In this study, effort was made to uncover the copy number variations of SD repeats by bioinformatic analysis and to detect changes in SD repeats during a plasmid-based assay, as a first step to understand its biological functions. The SD repeats were found to be mainly present in the cell surface proteins. The SD repeats were genetically unstable and polymorphic in terms of copy numbers and sequence compositions. Unlike SNPs, the change of its copy number was reversible, without frame shifting. More significantly, a rearrangement hot spot, the ATTC/AGRT site, was found to be mainly responsible for the instability and reversibility of SD repeats. These characteristics of SD repeats may facilitate bacteria to respond to environmental changes, with low cost, low risk and high efficiency.

## Introduction

All bacteria face a challenge of maximizing their fitness in a constantly changing environment. The major mechanism through which organisms from the same bacterial species can adapt is microevolution, which includes horizontal gene transfer and mutations. Horizontal gene transfer is common among bacteria, even amongst very distantly-related ones. This process is thought to be a significant cause of increased drug resistance [1]. Mutations are the main sources of novel variations and are a primary force behind microevolution. Mutations can be further divided into single nucleotide polymorphisms (SNPs), indels, genome rearrangement, copy number variation and changes in tandem repeats (TRs, also known as satellite DNA) [2].

TRs commonly exist in eukaryotic and prokaryotic organisms and they are mutation-prone DNA. Based on the length of repeat units, TRs are classified as microsatellites (1 to 9 nt) or minisatellites (≥10 nt) [3]. Errors during replication make TRs unstable, generating changes in the number of repeat units that are 100 to 10,000 times more frequent than point mutations [4]. Unlike SNPs, copy number changes of TRs are usually reversible. Microsatellites are ubiquitous in eukaryotes and in prokaryotic genomes. They have been mostly found in locations of protein-coding genes or their untranslated regions, where some of them could provide adaptive functional variability [5]. Conversely, minisatellites are mostly found in eukaryotic genomes, but under-represented in prokaryotic genomes. Most of studies on minisatellites have been carried out on yeast and humans, due to their correlations to different genetic diseases [6], [7]. On the other hand, rearrangement of minisatellite TRs in prokaryotics is rarely studied and not clearly understood.

The genome of a bacterial species is composed of conserved core genes and variable accessory genes. The core genome includes genes common to all strains in a population and these core genes are involved in essential functions. On the contrary, accessory genes have been shown to play a key role in host adaptation to environment and they are the portion of the genome that is variably present among individual strains [8]. Mobile genetic elements, such as plasmids, transposons, insertion sequences, integrons, prophages, genomic islands, and pathogenicity islands, are parts of accessory genes [8], [9]. These mobile elements facilitate interspecies and intraspecies genetic exchange. They are a major contributor to species diversity and play an important role in the pathogenicity of bacteria.

Pathogenicity islands encode genes which contribute to the virulence of the respective pathogen. Typical examples are adherence factors, toxins, iron uptake systems, invasion factors and secretion systems. Many adhesins from *Staphylococci* are known to contain minisatellite SD repeats. SD repeats are a sub-set of VNTR (Variable Number of Tandem Repeats) and encode the amino acid pair of serine-asparatate, with an array of 18-nucleotide repeats, whose elements follow the consensus GAY TCN GAY TCN GAY AGY, where N is any base and Y is T or C [10]. SD repeats are present in a variable repetitive region of these adhesins [11], such as the R domain of clumping factor A (ClfA). Most SD repeats of surface proteins have similar primary structural organizations: an N-terminal signal sequence (S) followed by a ligand-binding domain, which is exposed on the surface of the bacterial cell; a variable repetitive region (R domain) between the binding domain and a C-terminal anchoring domain, which is composed of a wall-spanning region (W) and a membrane-spanning domain (M) [11]. Recently, SD repeats have been explored for genotyping [12], [13], due to their polymorphisms in the copy numbers. It is known that a certain number of SD repeats are required for functional expression of the ligand-binding domain of ClfA on the cell surface [14]. However, the biological significance of SD repeats remains elusive. It is known that microsatellites function as contingency loci, which are defined as regions of hypermutable DNA that mediate high-frequency, stochastic, heritable and genotypic switching [15]. Whether minisatellites could also act as contingency loci is still not clear. As a first step, we were interested to study whether the SD repeats could contribute to *Staphylococcal* adaptive evolution.

A plasmid-based assay system in *E. coli* has been widely used to study rearrangement of prokaryotic tandem repeats [16], [17]. With the advent of DNA sequencing and bioinformatics, it is now also possible to trace and compare the DNA changes between original repeats and their variants. In this study, we first found the high instability of the SD repeats in *clfA* of a *S. aureus* strain and ATTC/AGRT site as a rearrangement hot spot in a plasmid-based system. Next, we expanded our findings to all organisms available in the database to study the distribution of SD repeats by bioinformatic analysis. Based on these novel findings, we conclude that SD repeats in associated surface proteins may contribute to *Staphylococcal* microevolution in adaption to environmental fluctuations.

## Results

### SD repeats in ClfA of *Staphylococcus aureus* contain perfect consensus in the center and imperfect consensus at two sides at the DNA level

**Figure 1:**
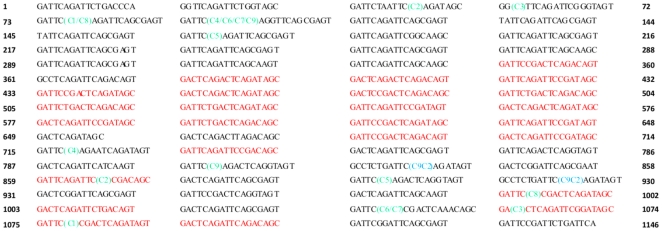
DNA sequence characterization of *clfA* SD repeats of *S. aureus* Smith Cp and the locations of rearranged sites from 9 variants of pNZ3004-ClfA.rRWM and 1 variant of pNZ3004-ClfA.SAR.rRWM.C9. The number at two sides represents the nucleotide number. The perfect consensus repeats are marked in red and the imperfect consensus repeats are marked in black. The repeat right after 15 central perfect consensus repeats has only 12 nucleotides, as this arrangement makes the whole repeats maximally conform the consensus. Each rearranged site in 9 variants of pNZ3004-ClfA.rRWM was marked by the name of variants in parentheses in green, with the region between them looped out during the rearrangement. For 1 variant of pNZ3004-ClfA.SAR.rRWM.C9, 5 SD repeats were added between the two arrangement sites and were not shown in the figure, marked by the name of the variant in parentheses in blue.

To characterize SD repeats in ClfA of *Staphylococcus aureus* at DNA level, the R domain of ClfA was amplified by using the primer F and primer R with the genomic DNA of *S. aureus* Smith Cp as template and the sequence was deposited in GenBank (accession number GU952273). As shown in Figure 1, there were a total 63 SD repeats in the R domain, with 25 SD repeats in red conforming exactly to the consensus GAY TCN GAY TCN GAY AGY. Among them, sixty percent (15 SD repeats) were located successively in the center. Other 38 repeats contained one or two triplets different from consensus, which did not encode Ser or Asp. Sixty-seven percent (30 out of 45) sites, where nucleotides did not follow the consensus, were located at the third triplet GAY of the consensus. Among them, 73.3% (22 of 30) were caused by GAY mutation to GCR, causing Asp mutation to Ala in the SD repeat array. The repeats containing “GCR” mutation were highly concentrated in the N terminal of the R domain and their sequences were very similar, implying that these “GCR” containing repeats could be formed by expansion rearrangements after initial point mutation. Further analyses of the DNA sequence of *clfA* SD repeats in 7 other published *S. aureus* strains confirmed the existence of this unique pattern: perfect consensus in the center and imperfect consensus at two sides (Table S1).

### A plasmid-based assay system in conjunction with sequencing reveals the high instability of SD repeats

To determine whether and how *S. aureus* with variable SD repeats initiate and process the rearrangement, a traceable plasmid-based assay in conjunction with rearranged DNA sequencing analysis was used. First, how cloning affects SD repeat stability was investigated, by cloning the RWM segment of ClfA from Smith Cp into pNZ3004. After cloning, nine positive colonies were randomly picked and DNA fragment containing SD repeat region was amplified by PCR. All PCR products showed that the SD repeats were shortened, in comparison with the original size of 1.5 kb on agarose gel electrophoresis. Further sequencing confirmed that repeat copy numbers were reduced during the cloning process, from 63 copies down to 8–29 repeats (Table 1). Moreover, analyses of 9 rearranged sites revealed several interesting aspects: (1): except for mutant pNZ3004-ClfA.rRWM.C3, a hot spot of rearrangement, ATTC/AGRT, was found in other 8 mutants. The broken ATTC then linked to downstream AGCC (or AGAC, CGAC) sites (Figure 1; Table 2). (2): none of the open reading frame of mutants was shifted after rearrangement. The recombination sites appeared within a repeat, not between repeats. (3): after rearrangement, 15 central perfect repeats were looped out, which greatly decreased the homogeneity and the length of SD repeats.

**Table 1:** Plasmids and their relevant features in this study.

Plasmid	Relevant features	Reference
pNZ3004	*E.coli-Lactobacillus* shuttle vector, pwv01 ori, Cmr, Emr; 4.9 kb	[37]
pNZ3004-ClfA.rRWM	RWM domains cloned into pNZ3004, SD repeats had rearrangements as indicated by ‘r’	this study
pNZ3004-ClfA.rRWM.C1	R domain contained 8 repeats	this study
pNZ3004-ClfA.rRWM.C2	R domain contained 18 repeats	this study
pNZ3004-ClfA.rRWM.C3	R domain contained 8 repeats	this study
pNZ3004-ClfA.rRWM.C4	R domain contained 29 repeats	this study
pNZ3004-ClfA.rRWM.C5	R domain contained 23 repeats	this study
pNZ3004-ClfA.rRWM.C6	R domain contained 11 repeats	this study
pNZ3004-ClfA.rRWM.C7	R domain contained 11 repeats	this study
pNZ3004-ClfA.rRWM.C8	R domain contained 13 repeats	this study
pNZ3004-ClfA.rRWM.C9	R domain contained 24 repeats	this study
pNZ3004-ClfA.SA.rRWM.C4	SA segment cloned into pNZ3004-ClfA.rRWM.C4	this study
pNZ3004-ClfA.SA.rRWM.C6	SA segment cloned into pNZ3004-ClfA.rRWM.C6	this study
pNZ3004-ClfA.SA.rRWM.C8	SA segment cloned into pNZ3004-ClfA.rRWM.C8	this study
pNZ3004-ClfA.SA.rRWM.C9	SA segment cloned into pNZ3004-ClfA.rRWM.C9	this study
pNZ3004-ClfA.SAR.rRWM.C9	SAR segment cloned into pNZ3004-ClfA.rRWM.C9	this study
pNZ3004-ClfA.SAR.rRWM.C9.C1^A^	87 repeats, instability of SD repeats discovered after 3 rounds of propagation	this study
pNZ3004-ClfA.SAR.rRWM.C9.C2	92 repeats, expansion of 5 SD repeats at rRWM segment	this study
pNZ3004-ClfA.SAR.rRWM.C9.C7^A^	87 repeats, instability of SD repeats discovered after one time of transformation	this study
pNZ3004-ClfA.SAR.rRWM.C9.C7.C1	deletion in SD repeats between SAR and rRWM, lost *Bam*HI site	this study
pNZ3004-ClfA.SAR.rRWM.C9.C7.C2	deletion in SD repeats between SAR and rRWM, lost *Bam*HI site	this study
pNZ3004-ClfA.SAR.rRWM.C9.C7.C3	deletion in SD repeats between SAR and rRWM, lost *Bam*HI site	this study
pNZ3004-ClfA.SAR.rRWM.C9.C7.C4	deletion in SD repeats between SAR and rRWM, lost *Bam*HI site	this study
pNZ3004-ClfA.SAR.rRWM.C9.C7.C5	deletion in SD repeats between SAR and rRWM, lost *Bam*HI site	this study
pNZ3004-ClfA.SAR.rRWM.C9.C7.C6	deletion in SD repeats between SAR and rRWM, lost *Bam*HI site	this study
pNZ3004-ClfA.SAR.rRWM.C9.C7.C7	SAR segment increase, may have expansion in SD repeats of SAR	this study
pNZ3004-ClfA.SAR.rRWM.C9.C7.C8	deletion in SD repeats of SAR segment	this study
pNZ3004-ClfA.SAR.rRWM.C9.C7.C10	deletion in SD repeats between SAR and rRWM, lost *Ba*mHI site	this study

A : The copy numbers of the SD repeats were same for the following plasmids: pNZ3004-ClfA.SAR.rRWM.C9.C1 and pNZ3004-ClfA.SAR.rRWM.C9.C3 to pNZ3004-ClfA.SAR.rRWM.C9.C10.

**Table 2:** The 5′ recombination sites in SD repeat regions of different variants.

Variants	Rearrangement site^A^
pNZ3004-ClfA.rRWM.C1	GATTC/AGAT
pNZ3004-ClfA.rRWM.C2	ATTC/AGAT
pNZ3004-ClfA.rRWM.C4	GATTC/AGGT
pNZ3004-ClfA.rRWM.C5	TCAGATTCAGCGAGTGATTC/AGAT
pNZ3004-ClfA.rRWM.C6	TCAGATTCAGCGAGTGATTC/AGGT
pNZ3004-ClfA.rRWM.C7	TCAGATTCAGCGAGTGATTC/AGGT
pNZ3004-ClfA.rRWM.C8	AGTGATTC/AGAT
pNZ3004-ClfA.rRWM.C9	AGTGATTC/AGGT
pNZ3004-ClfA.SAR.rRWM.C9.C2	GATTCAGACTCAGGTAGTGCCTCTGATTC/AGAT
pNZ3004-ClfA.SAR.rRWM.C9.C7.C1	AGCGATTC/AGAT
pNZ3004-ClfA.SAR.rRWM.C9.C7.C2	TTCAGCGAGTGATTC/AGAT
pNZ3004-ClfA.SAR.rRWM.C9.C7.C3	GATTC/AGAT
pNZ3004-ClfA.SAR.rRWM.C9.C7.C4	GATTCAGCGAGTGATTC/AGAT
pNZ3004-ClfA.SAR.rRWM.C9.C7.C5	ATTCTGGTAGCGATTCTAATTC/AGAT
pNZ3004-ClfA.SAR.rRWM.C9.C7.C6	GATTC/AGGT
pNZ3004-ClfA.SAR.rRWM.C9.C7.C8	AGTGATTC/AGGT
pNZ3004-ClfA.SAR.rRWM.C9.C7.C10	ATAG/CGAT
pNZ3004-ClfA.rRWM.C3	AGCGG B /TTCA

A : The DNA before slash is homologous sequence located at 5′ of two recombination sites of the mutant.

B : The nucleotides at 5′ of two recombination sites are not totally homologous in this mutant.

Next, effects of inserting additional R domain and the orientation of an insertion on rearrangement were examined, by cloning the segment SAR into pNZ3004-ClfA.rRWM.C9 to construct pNZ3004-ClfA.SAR.rRWM.C9. The R region of SAR segment was inserted into the vector at 3′, which was around 1.6 kb away from the 5′ insert site. After transformation, 10 positive colonies were randomly picked and extracted plasmids DNA were sequenced. Interestingly, nine colonies contained 87 copies of SD repeats, coming from original 63 copies from the SAR segment and 24 copies from the rRWM segment in pNZ3004-ClfA.rRWM.C9. However, pNZ3004-ClfA.rRWM.C9.C2 contained 5 additional SD repeats in the rRWM segment, indicating that SD repeat change was reversible (Figure 1; Table 2). It appears that the rearrangement of SD repeats only happened in the R region of RWM segment, not in the SAR segment. The R region of the RWM segment was close to the 5′ insert site, but away from the 3′ insert site; On the other hand, the R region of the SAR segment was close to the 3′ insert site, but away from the 5′ insert site. Thus, it seems that the SD repeat rearrangement was location and direction dependent.

### Plasmids containing high copies of SD repeats show high instability during propagation and transformation

**Figure 2:**
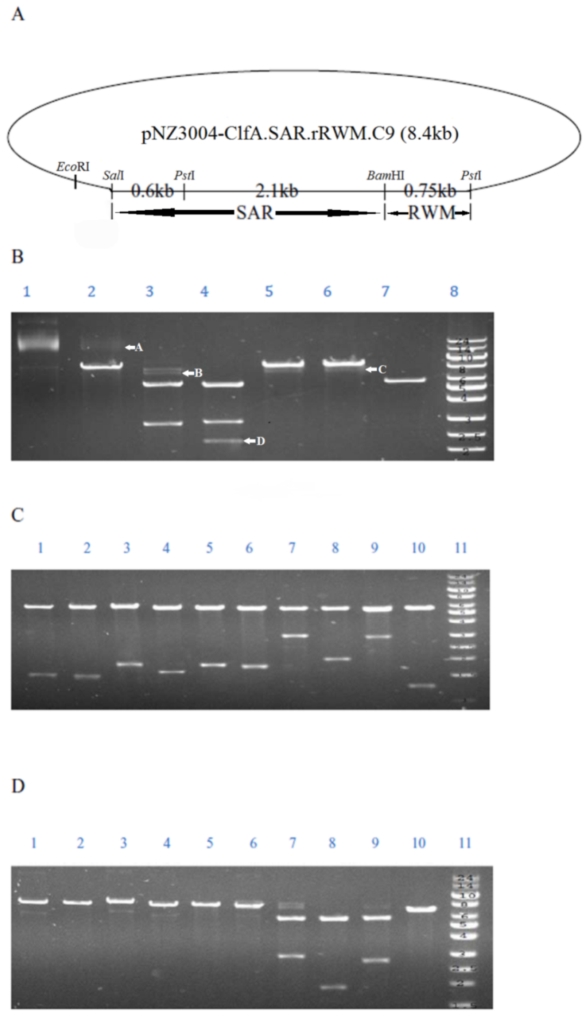
Instability of SD repeats shown by restriction enzyme analyses. (A) pNZ3004-ClfA.SAR.rRWM.C9 plasmid map showed locations of the restriction enzymes and sizes of different segments. (B) After 3 rounds of propagation, instability of constructs pNZ3004-ClfA.SAR.rRWM.C9.C1 was detected by restriction enzyme analyses. Lane 1: Uncut plasmid; Lane 2: Digested by *Bam*HI; Lane 3: Digested by *Bam*HI/*Sal*I; Lane 4: Digested by *Pst*I; Lane 5: Digested by *Sal*I; Lane 6: Digested by *Eco*RI; Lane 7: *Bam*HI restriction enzyme activity control. Plasmid PF01 digested by *Bam*HI; Lane 8: ultraRanger 1 kb DNA ladder (Kb). Arrows A and B indicated the incomplete digestion by *Bam*HI and the failed double digestion by *Bam*HI/*Sal*I, respectively. Arrow C showed the mixture of the shorter plasmid DNA. Arrow D revealed the rearrangement in the region between two *Pst*I sites. (C) The rearranged variants from the transformants of pNZ3004-ClfA.SAR.rRWM.C9.C7 digested by *Pst*I. Lane 1: pNZ3004-ClfA.SAR.rRWM.C9.C7.C1 (deletion in SD repeats between SAR and rRWM). Lane 2: pNZ3004-ClfA.SAR.rRWM.C9.C7.C2 (deletion in SD repeats between SAR and rRWM). Lane 3: pNZ3004-ClfA.SAR.rRWM.C9.C7.C3 (deletion in SD repeats between SAR and rRWM). Lane 4: pNZ3004-ClfA.SAR.rRWM.C9.C7.C4 (deletion in SD repeats between SAR and rRWM). Lane 5: pNZ3004-ClfA.SAR.rRWM.C9.C7.C5 (deletion in SD repeats between SAR and rRWM). Lane 6: pNZ3004-ClfA.SAR.rRWM.C9.C7.C6 (deletion in SD repeats between SAR and rRWM). Lane 7: pNZ3004-ClfA.SAR.rRWM.C9.C7.C7 (expansion in SD repeats between SAR and rRWM). Lane 8: pNZ3004-ClfA.SAR.rRWM.C9.C7.C8 (deletion in SD repeats between SAR and rRWM). Lane 9: pNZ3004-ClfA.SAR.rRWM.C9.C7.C9 (no rearrangement). Lane10: pNZ3004-ClfA.SAR.rRWM.C9.C7.C10 (deletion in SD repeats between SAR and rRWM). (D) The rearranged variants from the transformants of pNZ3004-ClfA.SAR.rRWM.C9.C7 double-digested by *Bam*HI and *Sal*I. Lane 1: pNZ3004-ClfA.SAR.rRWM.C9.C7.C1 (rearrangement occurred between SAR and rRWM, lost *Bam*HI site). Lane 2: pNZ3004-ClfA.SAR.rRWM.C9.C7.C2 (rearrangement occurred between SAR and rRWM, lost *Bam*HI site). Lane 3: pNZ3004-ClfA.SAR.rRWM.C9.C7.C3 (rearrangement occurred between SAR and rRWM, lost *Bam*HI site). Lane 4: pNZ3004-ClfA.SAR.rRWM.C9.C7.C4 (rearrangement occurred between SAR and rRWM, lost *Bam*HI site). Lane 5: pNZ3004-ClfA.SAR.rRWM.C9.C7.C5 (rearrangement occurred between SAR and rRWM, lost *Bam*HI site). Lane 6: pNZ3004-ClfA.SAR.rRWM.C9.C7.C6 (rearrangement occurred between SAR and rRWM, lost *Bam*HI site). Lane 7: pNZ3004-ClfA.SAR.rRWM.C9.C7.C7 (SAR segment increase, may have expansion in SD repeats of SAR). Lane 8: pNZ3004-ClfA.SAR.rRWM.C9.C7.C8 (deletion in SD repeats of SAR). Lane 9: pNZ3004-ClfA.SAR.rRWM.C9.C7.C9 (no rearrangement). Lane10: pNZ3004-ClfA.SAR.rRWM.C9.C7.C10 (rearrangement occurred between SAR and rRWM, lost *Bam*HI site). Lane 11: ultraRanger 1 kb DNA ladder (Kb).

It has been reported that propagation and transformation processes affect the sequence stability of repeats in plasmids [18]. Whether this was applied to the R repeat was subsequently studied. The *E. coli* containing pNZ3004-ClfA.SAR.rRWM.C9.C1 was propagated three rounds before the restriction enzyme analysis. The size of pNZ3004-ClfA.SAR.rRWM.C9.C1 was 8.4 kb, as shown in Figure 2A. The size of segment SAR was 2.7 kb, which was between *Sal*I and *Bam*HI. The whole SD repeat region was located in the segment between 2 *Pst*I sites with the size of 2.85 kb. *Bam*HI was located in the middle of the SD repeats region. After three rounds of propagation, the plasmid pNZ3004-ClfA.SAR.rRWM.C9.C1 was digested by *Bam*HI or double digested by *Bam*HI/*Sal*I, separately (Figure 2B). The incomplete *Bam*HI digestion in lane 2 and the failure of complete double digestion in lane 3 demonstrated occurrences of SD repeat rearrangements during the replications, with some rearranged DNA losing the *Bam*HI site. Similarly, the weak bands below the band of 8.4 kb in lanes 5 and 6 appeared when the plasmids were digested by unique restriction enzyme *Eco*RI or *Sal*I, separately, supporting the notion of SD repeat rearrangement. When digested by *Pst*I as shown in lane 4, there were 2 strong bands of 5.6 kb and 2.85 kb and some weak bands below 2.85 kb. These further indicated that rearrangement happened in the region between two *Pst*I sites.

**Figure 3:**
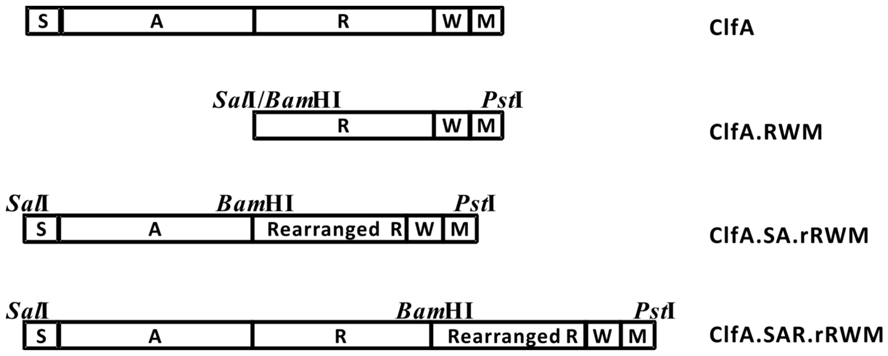
A schematic diagram of *S. aureus* ClfA organization and the insert fragments in the constructs. S: the signal sequence; A: the binding domain; R: the SD repeat region; W: the wall-spanning region; M: the membrane-spanning region. ClfA.RWM: the segment RWM of ClfA from *S. aureus* Smith Cp which was cloned into pNZ3004. ClfA.SA.rRWM: the inserted portion of pNZ3004-ClfA.SA.rRWM after cloning the segment SA of ClfA from *S. aureus* Smith Cp cloned into pNZ3004-ClfA.rRWM. ClfA.SAR.rRWM: the inserted portion of pNZ3004-ClfA.SAR.rRWM after cloning the segment SAR of ClfA from *S. aureus* Smith Cp into pNZ3004-ClfA.rRWM.

The construct pNZ3004-ClfA.SAR.rRWM.C9.C7 was used to study the instability during the transformation. It had 63 repeats in the SAR region and 24 repeats in the RWM region. After the plasmid was transformed into DH5α, 30 colonies were randomly picked and the plasmid DNA was extracted. According to PCR and sequencing data, 9 out of 30 had different sizes of SD repeats in comparison with the original pNZ3004-ClfA.SAR.rRWM.C9.C7. The restriction enzyme analysis results from the transformant variants were shown in Figure 2C and 2D. Among the 9 rearrangements, 8 transformants had deletion in the SD repeats, since the size between 2 *Pst*I sites was decreased (Figure 2C) with 7 transformants losing *Bam*HI sites (Figure 2D). One transformant had expansion in SD repeats of SAR region, as it showed a bigger SAR segment in the lane of agarose gel (Figure 2D, lane 7) than the control lane 9. All the restriction enzyme results were confirmed by sequencing results. In addition, 7 out of 8 deletions had ATTC/AGRT as the rearrangement sites like pNZ3004-ClfA.rRWM (Figure 3 and Table 2). While the instability of the plasmid pNZ3004-ClfA.SAR.rRWM.C9 containing 87 copies of SD repeats was observed after three rounds of propagation or one round of transformation, the plasmids pNZ3004-ClfA.SA.rRWM or pNZ3004-ClfA.rRWM mutants containing low copy numbers (8 to 29 copies) of SD repeats were stable after three rounds of propagation or one round of transformation. The results strongly indicated that the SD repeats with high copy numbers were less stable in the SD repeat-containing region.

### Nearly all the long SD repeats-containing proteins are the surface proteins of *Staphylococcus aureus* and *Staphylococcus epidermidis*

The results above showed the instability of the SD repeats under the laboratory condition. Therefore, it is plausible that such a variation also exists naturally in SD repeats in various organisms. Hence, the distribution of SD repeats was first explored at the protein level in this study. The blast from the Reference proteins database, the Swissprot database and the Non-redundant database yielded 94, 87 and 95 proteins containing SD repeats, respectively. Same proteins with different names have been identified by comparing their sequences with each other to avoid duplications.

The total copy numbers of SD repeats in all these identified proteins were counted and sorted by their sources. The copy numbers of SD repeats in proteins from non-bacterial sources were all less than 4 repeats. Thus, these proteins were not considered further. The distribution of 192 proteins containing SD repeats is presented in Tables 3 and S2. Among them, 155 proteins were from 44 different *S. aureus* strains (6 different types of proteins), 22 proteins from 12 different *S. epidermidis* strains (3 different types of proteins), and the other 15 proteins from 8 other bacteria. All these proteins were cell surface proteins of bacteria. In particular, 80.7% and 11.5% of them were from *S. aureus* and *S. epidermidis*, respectively.

**Table 3:** SD repeat variations in the surface proteins of different bacteria strains.

Strain	Protein name (No.)^A^	Repeat range			Variation site^B^		Variation distribution^C^			
		Smallest	Largest	Average	D	S	N	M	C	W
*S. aureus*	ClfA (36)	45	65	52.18	A,G,E,T,N	N,L	10–20		10–15	
*S. aureus*	ClfB (30)	9.33	59	37.75	G,E,N	N	5–10	1–5		
*S. aureus*	SdrC (28)	5	58.67	27.80	E	N,T	1–5	1–4		
*S. aureus*	SdrD (27)	22	38.33	30.53	E	C			0–5	
*S. aureus*	SdrE (28)	13.33	27.67	23.73	E		0–3			
*S. aureus*	Pls (6)	26.33	51.33	38.05	E	A				2–30
*S. epidermidis*	SdrF (4)	32.67	93	69.75	E	A,Y				12–25
*S. epidermidis*	SdrG (9)	9.33	33.33	22.63	A,G	N		1–5	1–5	
*S. epidermidis*	SdrH (9)	15	21	18.81	G	A,N		1–5	1–5	
*S. lugdunensis*	Fbl (4)	15.67	43.67	36.25	G,N	A				17–47
*S. haemolyticus*	Sdr (1)	29	29	29		A	2		29	
*S. saprophyticus*	SdrI (1)	142.67	142.67	142.67		A				111
*S. capitis*	SdrX (1)	34.67	34.67	34.67	G,E,C	N,L	5		8	
*S. caprae*	SdrZ (1)	21.33	21.33	21.33	G,N	A,Q				21
*L. plantarum*	Sdr (4)	81	267.67	181.92	G,N	A,G	2–3		2–3	
*A. baumannii*	Adhesin 1 (1)	235.67	235.67	235.67			None			
*A. baumannii*	Adhesin 2 (1)	167.67	167.67	167.67		A		2		
*K. pneumoniae*	Sdr (1)	424.33	424.33	424.33	E	A	2	14		1

A :The detected proteins available in the Reference, Swissprot and Non-redundant protein sequences database (accessed on August 3^rd^, 2011). The number in the parentheses is the number of strains which contain same protein.

B :The variation site in the SD repeats, the letters represent amino acids single-letter codes (SLC).

C :The distribution and number of variation sites in the proteins. N, C, M and W represent the mutations in the N terminus, C terminus, middle, or whole sequences of the SD repeats, respectively.

Among the 192 bacterial proteins containing SD repeats, the longest one was from *Klebsiella pneumonia* strain MGH 78578, with 424.33 copy number of SD repeats, while the shortest SD repeats containing protein was from the SdrC protein of *S. aureus* strain ST398, with only 5 SD repeats. The proteins with more than 70 copies of SD repeats all came from non-*S. aureus* strains. Similarly, the proteins with more than 100 copies of SD repeats were all distributed in non-*S. aureus* and non-*S. epidermidis* strains (Table 3 and Table S2). The numbers of SD repeats in proteins from strains *Acinetobacter baumannii*, *Lactobacillus plantarum*, *K. pneumonia* and *Staphylococcus saprophyticus* were all over 80. Interestingly, the SdrI protein from *S. saprophyticus* contained 142 copies of SD repeats. However, the Asp is replaced by Ala in the whole SD repeats sequence.

### Most sequence variations of SD repeats in *S. aureus* surface proteins occur at both ends

The variations of SD repeats in different surface proteins were further examined in different *S. aureus* strains (Table 3 and Table S2). Most variations for ClfA were observed at both 5′ and 3′ ends. The replacements of Asp were mostly by Ala, Gly, or Glu and a few by Thr or Asn. The replacements of Ser were mostly by Asn or a few by Leu. Variations for ClfB SD repeats were located mostly at 5′ end with a few in the middle. The most variation bias was Asp to Glu, Asn or Gly, and a few Ser was replaced by Leu. SD repeats in SdrC, SdrD and SdrE had variable copy numbers and less variation. A few variations for SdrC appeared at 3′ and in the middle, with the change of Asp to Glu and Ser to Asn or Thr. A few variations for SdrD were found at Ser to Arg and Asp to Glu. Very few Asp to Glu replacement in the SD repeats was found in SdrE in the 28 strains. Plasmin-sensitive surface proteins contained highly imperfect SD repeats in three different *S. aureus* strains, (Table 3 and Table S2). There were many variations from Ser to Ala through their whole sequences. Moreover, the repeats copy numbers of most SD repeats containing proteins in *S. aureus* were always close to the average value in a same protein family (Table 3 and Table S2). Despite the copy number of SD repeats in each surface protein showing a wide range, the average copy number of all surface proteins in a strain is similar, usually between 30–40 repeats (Table S2), suggesting possible existence of a “repeat number balance” in different SD repeat containing proteins in a single strain.

## Discussion

Minisatellites in prokaryotes could have their own characteristics different from those in eukaryotes as regard to instability mechanisms. ClfA contains the minisatellite SD repeats and this was used in this study as a model system to study repeat rearrangement mechanisms of minisatellites in prokaryotes. Our results revealed that the SD repeats were genetically unstable and polymorphic in terms of copy numbers and sequence compositions. Another novel finding from this study was the revelation of a rearrangement hot spot, the ATTC/AGRT site, which was found to be mainly responsible for the instability and reversibility of SD repeats. Lastly, we found that the proteins containing SD repeats were mainly present in the cell surface. All these results suggest that change of repeat numbers can be used as a means to adapt to environmental stress.

The first major finding from this study was genetical instability and polymorphism of the SD repeats. These were in agreement with our previous typing results [13], [19], [20]. In the process of constructing plasmids containing SD repeats, the instability of SD repeats was revealed, with the original copy number of 63 copies of SD repeats down to 8–29 copies and with the 15 perfect repeats in the center being looped out. In addition to the cloning, transformation and propagation increased the instability of SD repeats, which is in agreement with the previous reports on other minisatellites [6], [18]. The instability of SD repeats during the cloning process may be caused by the repair of DNA damage, which often occurs to the genome of *S. aureus*. In addition, some genes may be transferred among different strains by horizontal gene transfer. Indeed, we have found the *sdr* genes may be horizontally transferred among *S. aureus* [21]. Therefore, cloning, transformation and propagation represent different types of environmental stresses to bacteria. Our experimental results also showed that repeat copy numbers had influence on SD repeats stability. Plasmids containing high copy numbers of SD repeats were very unstable during propagation and transformation, while plasmids containing low copy numbers of SD repeats were very stable during same treatments.

After analysing the rearranged sequences, an ATTC/AGRT site was found to be mostly responsible for SD repeat rearrangement, including contraction in most cases and expansion in one case (Figure 1). The open reading frames of all rearranged sequences were not shifted and the rearrangements always occurred within repeats, leading to change of copy numbers but without affecting the intact structure of proteins containing SD repeats. These changes could be explained by the uniqueness of SD repeats sequence. The DNA sequence of SD repeats is GAY TCN GAY TCN GAY AGY. Thus, the ATTC sites only appear when the sequence is GAT TCN GAT TCN GAY AGY. Hence, if the sequence is rearranged, regardless of which base appears after the ATTC site after rearrangement, the open reading frame will never be shifted and does not produce a stop codon. In addition, it explains why plasmids containing high copy numbers of SD repeats were very unstable, since more repeats mean more ATTC/AGRT rearrangement sites, and more opportunities to rearrange the sequence. Besides the ATTC/AGRT rearrangement site, the second rearrangement site was also found in one case, the ATAG/CGAT site in clone pNZ3004-ClfA.SAR.rRWM.C9.C7.C10. Unlike the ATTC/AGRT rearrangement occurred in the first SD and the second SD site, the ATAG/CGAT rearrangement occurred in the third SD site, the GAY AGY site with GAT AG/C actually. It has been proposed that the primordial codon for serine was TCN, while the codon AGY appeared later [22], which explained a significant higher proportion of serine resides being coded by TCN, rather than by AGY. Considering this finding, it seems that AGY was the end signal of SDSDSD repeat and it maintains the units intact. Further, the rearrangement mostly occurred in the GAY TCN code, much less in the GAY AGY code, suggesting that AGY was less prone to mutation than TCN.

Rearrangement of SD repeats could be through models of replication slippage [23], which caused the segment of DNA among the repeats to be “loop-out” or expanded. The rearrangement might also be initiated by DNA repair induced slippage. TRs could form barriers for DNA polymerase III because of their tendency to form secondary structures [24], [25], which might induce the slipped-strand mispairing (SSM). This type of mechanism has been reported to occur widely for short microsatellite TR [23]. It appears that the same mechanism can also be applied to minisatellite SD repeats. When the RWM fragment was inserted, the rearrangement occurred in the R region. However, when the SAR fragment was cloned into vector pNZ3004-ClfA.rRWM, we did not detect the rearrangement of the R domain in the SAR fragment, suggesting that DNA repair mispairing, rather than DNA replication mispairing, was responsible for the rearrangement. DNA mispairing induced rearrangement can only happen at the downstream of the insertion, not at the upstream of the insertion. Though a RecA-independent crossover could also induce rearrangement of repeat sequences in bacteria [26], all mutants in this study were produced from just one cloning transformation and each positive colony contained only one type of variant, implying the RecA-independent crossover was impossible.

The distribution of SD repeats in all organisms was also investigated to analyze its potential multi-phenotypes. All proteins containing over 4 copies of SD repeats were of bacterial origins. Among them, over 90% were from *S. aureus* and *S. epidermidis*. *S. aureus* and *S. epidermidis* are the most common and devastating pathogens among *Staphylococcal* species. However, the longest SD repeats in them were only 65 and 93, respectively. It was also found that small oscillations in repeat numbers around the average number were the rule for each locus in genetically distinct strains (Table 3 and Table S2). All these proteins containing SD repeats are adhesins and they are redundant and complimentary [27]. Stochastic switching of six SD repeat loci in *S. aureus*, each generating only two genotypes, can potentially generate up to 64 phenotypes. Hence, it may not be necessary for any of the six loci in *S. aureus* to evolve a very long SD repeat, still quickly responding to environmental fluctuations. Mechanisms facilitating rapid phenotypic adaptation include: (1) built-in regulatory mechanisms that allow individual bacteria to alter gene expression in response to new environments [28], (2) import of DNA from other strains that are already adapted to the current environment [1], [21], and (3) “contingency loci” that mutate rapidly, creating phenotypic variation amongst bacteria that are otherwise genetically identical [15]. The built-in regulatory mechanisms will respond quickly to the environment fluctuation, but the genotype is not changed and it may not be quite enough for bacteria to survive well in a long-time range. Although there are clear fitness advantages to an organism to import DNA from other strains, however, there are several drawbacks for this strategy [29]. Hence, contingency loci, which act as the third strategy, have been placed on the spotlight for the rapid phenotypic adaptation. Recently, accumulating evidence has supported the hypothesis that some TRs could have a positive role in adaptive evolution [30]. For example, some microsatellite sequences found in *Haemophilus influencae* and *Neisseria meningitidis* function as contingency loci [15], [31]. However, there has not been any direct evidence that minisatellite sequences can also act as contingency loci in bacteria.

Our results suggest that variation of SD repeats may facilitate bacteria, especially in *Staphylococci*, to respond to environmental changes, with low cost, low risk and high efficiency. Firstly, the perfect ATTC/AGRT rearrangement sites in the GAY TCN GAY TCN GAY AGY sequence qualify the SD repeats as a very safe fine tuning site, which changes the length but avoids a frame-shift mutation totally. This feature is essential to keep an anchoring domain's stability, thus maintaining the function of the associated surface proteins containing SD repeats. Addition or deletion of repeat units in individuals of a population can cause potential functional diversity to adapt to environmental change immediately. Secondly, the SD repeats were very unstable in the process of cloning, transformation and propagation, suggested it will respond very quickly to the rapid environment fluctuation. Thirdly, the structure of SD repeats showed a high disorder propensity (data not shown). This high disorder structure could make SD repeats a very flexible linker to attach an anchoring domain with a binding domain, and function as a hinge. SD repeat length variation can cause fine turning of surface structures or change position/location of the binding domain for specific interaction with ligands, or with the host immune system. Fourthly, our findings that most proteins containing SD repeats were adhesins is in agreement with the notion that contingency loci are mostly found within cell surface genes involved in cell rescue, defence and virulence [32], [33]. Strongly biased distribution of SD repeats in the surface proteins of *Staphylococci* suggested that SD repeats are subject to strong selection. *Staphylococcal* surface proteins play an important role in the interaction with host cells. The infection process of *Staphylococcal* pathogens is initially through adherence to host tissue, which is mediated by the *Staphylococcal* surface proteins called microbial surface components recognizing adhesive matrix molecules (MSCRAMMS) that specifically bind to host extracellular matrix (ECM) components such as collagen, fibrinogen/fibrin and fibronectin [11]. The MSCRAMMS containing SD repeats can act as virulence factors to mediate adhesion and invasion, and have a role in evasion of the host immune system [34], [35]. However, the linkages between phenotypic changes and the changes of SD repeats need to be confirmed by further experiments.

Our results have shown that most proteins containing SD repeats are adhesins. In addition, SD repeats undergo high rates of length variation through slippage in an in vitro system. Thus, variation of SD repeats in bacterial membrane proteins could be a strategy by bacteria to modulate the structure of associated surface proteinsand consequently affects protein-host interactions in different host cells and environment. The novel insight into the importance of SD repeats will lead to a better understanding of staphylococcal pathogenesis evolution and may provide a potential countermeasure for staphylococcal infection.

## Materials and Methods

### Bacterial strains and growth conditions

RecA-independent *E. coli* DH5α was used as a cloning strain. It was cultured at 37°C in Luria-Bertani medium. For plasmid-harboured strains, 10 µg/ml of chloramphenicol was added in the medium. *S. aureus* Smith Cp was cultured at 37°C in a Nutrition Broth medium. Agar plates were prepared by addition of 1.5% or 2% agar to each medium.

### DNA extraction, manipulation and DNA sequencing

*S. aureus* Smith Cp genomic DNA was extracted as described by Hull et al. [36]. Plasmids were extracted using a Qiagen mini-prep or mid-prep kit (Qiagen, Mississauga, Ontario, Canada). PCR products were purified with a Qiagen PCR purification kit (Qiagen). All restriction enzymes were purchased from New England Biolab (Mississauga, Ontario, *Canada*). T4 DNA ligase, calf intestinal alkaline phosphatase (CIAP) and Taq polymerase were purchased from Invitrogen (Burlington, Ontario, Canada). DNA sequencing was carried out at the McGill University and Genome Quebec Innovation Centre.

### Plasmids and cloning strategy for construction of different variants

The plasmids used in this study are listed in Table 1. *E.coli/Lactobacillus* shuttle vector pNZ3004 is a low copy plasmid, and the copy number of pNZ3004 in *E. coli* is only 2.4 per cell [37]. It was used as a cloning vector and the genomic DNA of *S. aureus* Smith Cp was used as template. The schematic diagram of re-constructed ClfA in pNZ3004 is shown in Figure 3. Surface protein ClfA is composed of five domains: the signal sequence (S), the binding domain (A), the SD repeat region (R), the wall region (W) and the membrane-spanning region (M). The segment RWM containing 63 SD repeats was PCR amplified to generate restriction sites *Sal*I and *Bam*HI at 5′, *Pst*I at 3′, respectively, by primers F-545 and R-933 (all the primers are listed in Table 4). The PCR product and vector pNZ3004 were sequentially digested by *Sal*I and *Pst*I. The digested vector was treated with CIAP before ligation. After ligation and transformation, the positive colonies were screened by PCR and further confirmed through sequencing, using primers F-pNZ3004 and R-933.

**Table 4:** Primers used in this study.

Primer	Primer sequence (5′-3′)	
F	CCTGATGAGCCTGGTGAAAT	forward
F-1	ACGCGTCGACTATGAATATGAAGAAAAAAGAAAAACACGC	forward
F-505	TTACGTTCAACTTTATATGG	forward
F-545	ACGCGTCGACGGATCCCCTGAACAACCTGATGAGC	forward
F-pNZ3004	AGGAGGTAGTCCAAATGGC	forward
R	TTAGAACCTGACTCGGAATCG	reverse
R-226	CGGAATTCTTACGGTGCATCTGCAGCTAC	reverse
R-545	CGGGATCCAACAACTGGTTTATCGATACCGT	reverse
R-869	CGGGATCCATTGTTAGAACCTGACTCGG	reverse
R-933	AACTGCAGTTATTTCTTATCTTTATTTTCTTTTTTTCTTCTG	reverse

To construct pNZ3004-ClfA.SA.rRWM, the segment SA was PCR amplified to generate restriction sites *Sal*I at 5′ and *Bam*HI at 3′, respectively, by using primer F-1 and R-545, and cloned into vector pNZ3004-ClfA.rRWM. PCR Screening and sequencing were done by using primers F-pNZ3004 and R-933. Similarly, the segment SAR was PCR produced by using primers F-1 and R-869, and cloned into vector pNZ3004-ClfA.rRWM to construct pNZ3004-ClfA.SAR.rRWM. PCR screening was done by using primers F-pNZ3004 and R-226, and sequencing by using primers F-505 and R-933.

### The plasmid-based assay

For testing the stability of SD repeats in the constructed plasmids, plasmid-harbouring cells were scratched from glycerol stocks in a 5 ml LB liquid media for overnight culture as one round of propagation. The plasmids extracted after three rounds of propagation were used for restriction enzyme analyses and sequencing.

To test the effect of transformation on the stability of the SD repeats in constructed plasmids, approximately 100 ng of constructed plasmids were transformed into DH5α. Then the colonies were picked and cultured, and the plasmids were isolated by Miniprep as one round of transformation for sequencing and restriction enzyme analysis.

### Restriction enzyme analyses

Plasmids DNA were mixed well with 10×NEB 3 buffer, 10×BSA and an excess of appropriate restriction endonuclease (*Bam*HI, *Sal*I, *Pst*I, *Eco*RI or *Bam*HI/*Sal*I, 20–30 U per µg of DNA) at a final volume of 10 µl. Digestion was performed at 37°C for 4 hours before termination by adding 6× electrophoresis loading buffer. The samples were loaded on 1% agarose gel with ultrarange 1 kb DNA ladder as a size marker (Norgen, Toronto, Ontario, Canada).

### Bioinformatical analysis

**Figure 4:**
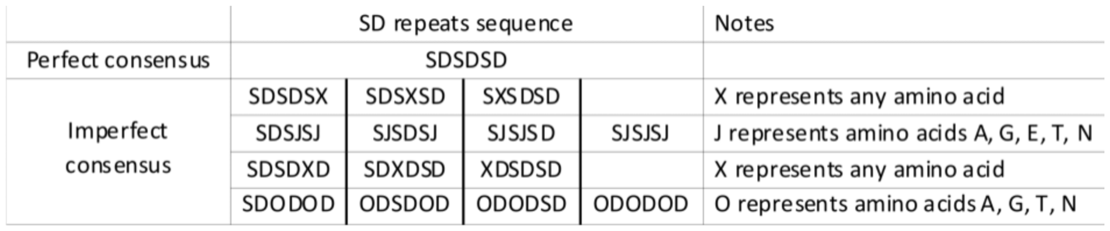
The definition of perfect and imperfect SD repeats. The TRs sequence “SDSDSD” was defined as a perfect consensus and an imperfect consensus was defined by the repeat contains 1–3 residues which did not follow the consensus “SDSDSD” sequence. Both perfect consensus and imperfect consensus repeats were counted as SD repeats.

In order to check the distribution of SD repeats, the NCBI protein blast software was used to screen proteins in all organisms in the Reference proteins database, the Swissprot protein sequences database and the Non-redundant protein sequences database (http://www.ncbi.nlm.nih.gov/BLAST/, accessed on August 3^rd^, 2011), the query amino acid sequence was SDSDSDSDSDSD. The blasting results from all three databases were sorted from the highest to the lowest by total score value, and the proteins with a total score value above 100 were included in our further analyses. The TRs sequence “SDSDSD” was defined as a perfect consensus and an imperfect consensus was defined by the repeat contains 1–3 residues which did not follow the consensus “SDSDSD” sequence, such as “SDSDSE”. Both perfect consensus and imperfect consensus repeats were counted as SD repeats. The detailed definition was shown in Figure 4. The variation was defined by the appearance of other residues in the repeat array instead of serine or aspartate. The copy number of SD repeats in detected proteins was calculated by using total residue number of repeats divided by 6. In addition, the DNA tandem repeat unit of the detected SD repeats was checked against the consensus GAY TCN GAY TCN GAY AGY for its variation.

## Supporting Information

Table S1 **Variations of ClfA SD repeats in 8** ***S. aureus*** **strains.** ^A^: Alignment of other strains with *S. aureus* Newman. ^B^: The perfect repeats which are located in the centre of the SD repeat region of proteins.
(DOC)

Table S2 **SD repeats variations in the surface proteins of different bacteria strains^A^.** ^A^: The detected proteins available in the Reference, Swissprot and Non-redundant protein sequences database (accessed on August 3^rd^, 2011). The number in the parentheses is the number of strains which contain same protein. ^B^: NA represents not available.
(DOC)
