# Supplementary material for: Variation of Serine-Aspartate Repeats in Membrane Proteins Possibly Contributes to Staphylococcal Microevolution
Source: PLoS One. 2012 Apr 11;7(4):e34756. doi: 10.1371/journal.pone.0034756 (PMC3324548; doi:10.1371/journal.pone.0034756)
Supplement: Table S1 — Variations of ClfA SD repeats in 8 S. aureus strains. A: Alignment of other strains with S. aureus Newman. B: The perfect repeats which are located in the centre of the SD repeat region of proteins. (DOC) [file pone.0034756.s001.doc]

**Table S1. Variations of ClfA SD repeats in 8 *S. aureus*** strains

| Strain | DNA sequence^A^ | Amino acids sequence^A^ | No. of perfect repeats (Centre ^B^) | GenBank ID |
| --- | --- | --- | --- | --- |
| Newman |  |  | 31(23) | Z18852.1 |
| COL | 100% | 100% | 31(23) | CP000046.1 |
| USA300 | 100% | 100% | 31(23) | CP000255.1 |
| MSSA476 | 92% | 97% | 28(13) | BX571857.1 |
| RF122 | 91% | 92% | 20(11) | AJ938182.1 |
| N315 | 86% | 88% | 31(25) | BA000018.3 |
| Smith. Cp | 83% | 88% | 25(15) | GU952273 |
| MRSA252 | 82% | 86% | 35(11) | BX571856.1 |

^A^: Alignment of other strains with *S. aureus* Newman.

^B^: The perfect repeats which are located in the centre of the SD repeat region of proteins.
