# Supplementary material for: Variation of Serine-Aspartate Repeats in Membrane Proteins Possibly Contributes to Staphylococcal Microevolution
Source: PLoS One. 2012 Apr 11;7(4):e34756. doi: 10.1371/journal.pone.0034756 (PMC3324548; doi:10.1371/journal.pone.0034756)
Supplement: Table S2 — SD repeats variations in the surface proteins of different bacteria strainsA. A: The detected proteins available in the Reference, Swissprot and Non-redundant protein sequences database (accessed on August 3rd, 2011). The number in the parentheses is the number of strains which contain same protein. B: NA represents not available. (DOC) [file pone.0034756.s002.doc]

**Table S2. SD repeats variations in the surface proteins of different bacteria strains ^A^**

| *S. aureus* | Strain | ClfA | ClfB | SdrC | SdrD | | SdrE | **Average** |
| --- | --- | --- | --- | --- | --- | --- | --- | --- |
|  | MRSA252 | 65.00 | 35.33 | 23.00 | NA^B^ | | 23.67 | **36.75** |
|  | NCTC8325 | 49.00 | 36.33 | 36.33 | 27.67 | | NA | **37.33** |
|  | TW20 | 49.00 | 59.00 | 24.33 | 33.00 | | 23.67 | **37.80** |
|  | Newman | 50.00 | 41.33 | 28.33 | 22.00 | | 27.67 | **33.87** |
|  | MSSA476 | 49.33 | 40.00 | 30.00 | 30.33 | | 24.33 | **34.80** |
|  | COL | 50.00 | 41.33 | 28.33 | 33.00 | | 27.67 | **36.07** |
|  | RF122 | 46.33 | 33.33 | 33.67 | NA | | 19.67 | **33.25** |
|  | ED98 | 58.33 | 36.33 | 33.67 | 33.67 | | 24.33 | **37.27** |
|  | ED133 | 49.33 | 41.67 | 31.67 | 23.67 | | 17.33 | **32.73** |
|  | 04-02981 | 45.33 | 36.33 | 29.33 | 33.67 | | 26.33 | **34.20** |
|  | N315 | 59.00 | 36.33 | 29.33 | 33.67 | | 24.33 | **36.53** |
|  | Mu50 | 50.00 | 36.33 | 29.33 | 33.67 | | 24.33 | **34.73** |
|  | Mu3 | 50.00 | 36.33 | 29.33 | 33.67 | | 24.33 | **34.73** |
|  | JKD6159 | 57.00 | 54.33 | 58.67 | 32.33 | | 25.33 | **45.53** |
|  | MW2 | 52.33 | 40.33 | 29.67 | 27.33 | | 24.33 | **34.80** |
|  | USA300-FPR3757 | 50.00 | 42.33 | 28.33 | 33.00 | | 25.67 | **35.87** |
|  | USA300-TCL1516 | 50.00 | 42.33 | 28.33 | 33.00 | | 25.67 | **35.87** |
|  | TCH130 | NA | NA | 29.33 | 33.67 | | 24.33 | **29.11** |
|  | JH1 | 45.00 | 36.33 | 31.33 | 25.67 | | 26.33 | **32.93** |
|  | JH9 | 45.00 | 36.33 | 31.33 | 25.67 | | 26.33 | **32.93** |
|  | ST398 | NA | NA | 5.00 | 33.67 | | NA | **19.34** |
|  | MN8 | 63.00 | 36.33 | 26.00 | 38.33 | | 23.67 | **37.47** |
|  | M876 | 61.67 | 44.33 | 32.00 | 30.33 | | 26.67 | **39.00** |
|  | A9781 | 45.33 | NA | 31.33 | NA | | NA | **38.33** |
|  | A9763 | 45.33 | 9.33 | NA | NA | | NA | **27.33** |
|  | A9719 | 45.00 | NA | NA | NA | | 13.33 | **29.17** |
|  | A9635 | 56.00 | NA | NA | NA | | NA | **56.00** |
|  | A9299 | 59.00 | NA | NA | 25.67 | | 17.33 | **34.00** |
|  | A6224 | 45.33 | 15.33 | NA | 25.67 | | NA | **28.78** |
|  | A5948 | 46.00 | NA | 26.33 | 25.00 | | NA | **32.44** |
|  | A8117 | NA | 36.33 | NA | NA | | NA | **36.33** |
|  | E1410 | 61.67 | 44.33 | 31.00 | NA | | NA | **45.67** |
|  | A6300 | 45.33 | 36.33 | 28.33 | NA | | 20.33 | **32.58** |
|  | 65-1322 | 61.67 | 37.33 | NA | NA | | NA | **49.50** |
|  | 55/2053 | 61.67 | NA | NA | NA | | NA | **61.67** |
|  | JKD6008 | 50.00 | 29.33 | NA | 32.00 | | 23.00 | **33.58** |
|  | Mu50-omega | 49.00 | 36.33 | NA | 33.67 | | 24.33 | **35.83** |
|  | WBG10049 | 60.00 | 45.33 | NA | NA | | 25.67 | **43.67** |
|  | ATCC51811 | 52.33 | NA | 30.67 | 31.33 | | 24.33 | **34.67** |
|  | **Average** | **52.18** | **37.75** | **27.80** | **30.53** | | **23.73** |  |
| *S. aureus* | Strain | Pls | | | | | | |
|  | 3172 | 38.33 | | | | | | |
|  | 3134 | 31.33 | | | | | | |
|  | c33s | 26.33 | | | | | | |
|  | COL | 51.33 | | | | | | |
|  | NCTC 10442 | 34.00 | | | | | | |
|  | No specific name | 47.00 | | | | | | |
|  | **Average** | **38.05** | | | | | | |
| *S. epidermidis* | Strain | SdrF | SdrG | SdrH | **Average** | | | |
|  | ATCC 9491 | 93.00 | NA | 20.00 | **56.50** | | | |
|  | ATCC 12228 | 76.33 | 30.00 | 19.00 | **41.78** | | | |
|  | VCU045 | 32.67 | 33.33 | NA | **33.00** | | | |
|  | SK135 | 77.00 | 9.33 | 15.00 | **33.78** | | | |
|  | RP62A | NA | 9.33 | 19.00 | **14.17** | | | |
|  | K28 | NA | 9.33 | NA | **9.33** | | | |
|  | W23144 | NA | 15.33 | 19.33 | **17.33** | | | |
|  | HB | NA | 36.00 | NA | **36.00** | | | |
|  | VCU037 | NA | 32.33 | 21.00 | **26.67** | | | |
|  | M23864:W2(grey) | NA | 28.67 | 21.00 | **24.84** | | | |
|  | VCU105 | NA | NA | 18.00 | **18.00** | | | |
|  | FRI909 | NA | NA | 17.00 | **17.00** | | | |
|  | **Average** | **69.75** | **22.63** | **18.81** |  | | | |
| *S. lugdunensis* | Strain | Fb1 | | | | | | |
|  | 2342 | 43.67 | | | | | | |
|  | HKU09-01 | 15.67 | | | | | | |
|  | M23590 | 41.00 | | | | | | |
|  | N920143 | 44.67 | | | | | | |
|  | **Average** | **36.25** | | | | | | |
| *S. haemolyticus* | Strain | Sdr | | | | | | |
|  | JCSC1435 | 29.00 | | | | | | |
| S. *saprophyticus* | Strain | SdrI | | | | | | |
|  | 7108 | 142.67 | | | | | | |
| *L. plantarum* | Strain | Sdr | | | | | | |
|  | ATCC14917 | 186.00 | | | | | | |
|  | JDM1 | 81.00 | | | | | | |
|  | ST-III | 217.00 | | | | | | |
|  | WCFS1 | 267.67 | | | | | | |
| *K. pneumoniae* | Strain | Sdr | | | | | | |
|  | MGH 78578 | 424.33 | | | | | | |
| *S. capitis* | Strain | SdrX | | | | | | |
|  | ATCC 49326 | 34.67 | | | | | | |
| *S. caprae* | Strain | SdrZ | | | | | | |
|  | 96007 | 21.33 | | | | | | |
| *A. baumannii* | Strain | Adhesin 1 | | | | Adhesin 2 | | |
|  | SDF | 235.67 | | | | 167.67 | | |

^A^: The detected proteins available in the Reference, Swissprot and Non-redundant protein sequences database (accessed on August 3^rd^, 2011). The number in the parentheses is the number of strains which contain same protein.

^B^: NA represents not available.
